# Primary characterization of the immune responses in Tibetan pigs infected with Chinese Tibet isolate of *Trichinella spiralis*

**DOI:** 10.1186/s12917-021-02806-z

**Published:** 2021-02-28

**Authors:** Li Tingting, Li Wenhui, Zhang Nianzhang, Qu Zigang, John A Ohiolei, Li Li, Yan Hongbin, Jia Wanzhong, Fu Baoquan

**Affiliations:** 1 State Key Laboratory of Veterinary Etiological Biology, Key Laboratory of Veterinary Parasitology of Gansu Province, Lanzhou Veterinary Research Institute, Chinese Academy of Agricultural Sciences, Lanzhou, China; 2 Jiangsu Co-innovation Center for the Prevention and Control of Important Animal Infectious Diseases and Zoonoses, Yangzhou University College of Veterinary Medicine, Yangzhou, China

**Keywords:** *Trichinella spiralis*, Tibetan pigs, Antibody, Cytokine

## Abstract

**Background:**

Trichinellosis, caused by *Trichinella spiralis*, is a serious foodborne parasitic zoonosis. Tibetan pig is an infrequent, endemic plateau pig species, mainly distributed in Tibet Plateau, China. Because of the free-range system, Tibetan pigs are at risk of infection with *Trichinella*. The present study aimed to primarily profile the characteristics of *T. spiralis* infection in Tibetan pigs, including IgG levels, larvae burdens, and cytokines.

**Results:**

The immune responses to Chinese Tibet *T. spiralis* isolate infection in Tibetan pigs with different doses were investigated in a tracking duration of 49 days. The muscle larvae per gram (lpg) were evaluated at 105 days post-infection (dpi). The results showed that the mean larval number of *T. spiralis* in Tibetan pigs increased with infective dose, with average lpg values of 3.5, 50.4 and 115.6 for Tibetan pigs infected with 200, 2,000, and 20,000 muscle larvae (ML) of *T. spiralis.* The anti-*Trichinella* IgG increased with inoculum dose and dpi, and peaked at 49 dpi. The kinetics of cytokines in the sera was detected by microarray, including interferon-γ (IFN-γ), interleukin (IL)-1β, IL-8, IL-12, IL-4, IL-6, IL-10, Granulocyte-macrophage Colony Stimulating Factor (GM-CSF), tumor necrosis factor (TNF)-α and transforming growth factor (TGF)-β1. The Th1/Th2 mixed cytokines were detectable in all samples. Interleukin-12 demonstrated the highest concentration compared to other cytokines and peaked at 42 dpi. Almost all cytokines were maintained at a high level at 42 dpi. Additionally, we also report a *Trichinella* seropositive rate of 43.9 % (18 out of 41) from field samples of Tibetan pigs.

**Conclusions:**

The present study showed an increased Th1/Th2 mixed cytokines in Tibetan pigs elicited by *T. spiralis*. The high seroprevalence of *Trichinella* infection in field samples of Tibetan pigs further raises serious concern for the prevention and control of trichinellosis in this host for public health safety.

## Background

Trichinellosis, a re-emerging food-borne parasitic zoonosis, is caused by nematodes of the genus *Trichinella* [1]. *Trichinella* shows a worldwide distribution because of its wide range of hosts, including humans and pigs [2]. Human infection is mainly caused by the consumption of raw or undercooked meat containing infective larvae of *Trichinella* [3]. It is estimated that approximately 11 million people are infected by *Trichinella* across the world [2]. Historically, pork and its products are regarded as the main sources of human trichinellosis [4]. However, recent reports on human trichinellosis have shown that human infection is more likely from the consumption of infected meat originating from wild carnivores and scavengers [5]. Many human outbreaks reported in recent years across Europe, Asia, and North and South America were reportedly due to consumption of wild boar meat which is currently the second most important source of human trichinellosis [5]. In addition to the public health problem of trichinellosis, the economic losses caused by *T. spiralis* are huge. Only during 1996, there were more than 500 million pigs slaughtered because of trichinellosis [6]; the Chinese government spent about $26 million on annual swine inspection. From 1964 to 2011, the cost of treating human cases was about $150 million [7].

Trichinellosis is also considered an emerging or re-emerging zoonotic disease in China, where approximately more than 40 million people could be at risk of infection [8]. The main endemic areas of human trichinellosis are Yunnan, Henan, Hubei, Guangxi, Tibet and northeastern China [8, 9]. Due to the predilection sites of *Trichinella* larva which occur in the diaphragm of pigs, detection is often restricted to postmortem inspection of carcasses. Meanwhile, pork is still considered the major source of outbreaks of trichinellosis in humans in China [4]. Surveys of *Trichinella* infection in pigs have been reported in Henan, Qinghai, Yunnan, Heilongjiang and some other provinces/autonomous regions in China [8–10]. Pig infection is acquired primarily through ingestion of infectious encysted *T. spiralis* muscle larvae (ML). After the ML are released in the stomach, they invade the intestinal epithelium and mature into adults (Ad). The newborn larvae (NBL) are shed by fertilized females at about day 5 after entering the epithelium. These NBL migrate via the lymphatics and penetrate the sarcolemmal membrane to form cysts. The immune response is characterized as a mixed Th1/Th2 response during the initial intestinal phase and switches into a predominance of Th2 response once the adults are matured [11].

*Trichinella* prevalence is well controlled in pigs under industrial farming conditions [4], whereas, the risk of parasite spillover continues in pigs under poor hygienic conditions, such as backyard or free-range systems [4, 6]. The Tibetan pig is a primitive Chinese native pig breed, which is distributed in the Tibet Plateau and the surrounding areas [7]. The meat of Tibetan pigs is a well-liked delicacy among locals and even residents in other regions of China due to its tender taste. However, due to the free-range system in which the pigs are raised, they are exposed to *Trichinella* and increases the risk of infection in humans. Nonetheless, there is still a paucity of information regarding *Trichinella* infection in Tibetan pigs.

To characterize *Trichinella* infection in Tibetan pigs, Tibetan pigs were infected with different doses of Chinese Tibet *T. spiralis* isolate and the dynamics of the immune response against the infection and the worm burden in different muscle tissues during the infection course were monitored. The results of this study represent the first profile on *T. spiralis* infection in Tibetan pigs, which improves our understanding of the immunology and pathology of *T. spiralis* in this swine species and also serves as baseline data for future studies.

## Results

### The muscle larvae burden of infected organs

The ML density in different muscle tissues of infected pigs is shown in Table 1. The result demonstrates a positive correlation between infective dose and the mean larval burden, with average lpg values of 3.5, 50.4 and 115.6 for Tibetan pigs infected with 200 ML, 2,000 ML, and 20,000 ML doses of *T. spiralis*. The correlation between the mean larval number and the infective dose is described by the equation: y = 0.0048x + 20.722 (R^2^ = 0.8841). The highest burdened of ML after different doses of *T. spiralis* infection were found in the diaphragm and tongue of Tibetan pigs, which were identified as the susceptible tissues. The minimum larvae load was found in the intercostal muscles of the pigs after infection with 20,000 or 200 larvae 105 dpi, and in the masseter after infection with 2000 larvae (Table 1).

**Table 1:** The larvae burden in Tibetan pigs infected with different doses of Chinese Tibet isolate of *T.spiralis*

lpg (larvae per g of muscle)	200 ML	2,000 ML	20,000 ML
diaphragm	9.9	59.9	315
tongue	5.2	105.9	130
masseter	3.4	22	95
intercostal	0.7	53.3	52.3
psoas	1	32	61.5
gluteus	3.2	38.3	88.8
foreleg	2.6	48.5	104.8
hind leg	2.1	43.2	77.4
Mean	3.5	50.4	115.6

### Anti-Trichinella-IgG kinetics in sera of infected Tibetan pigs

**Fig. 1:**
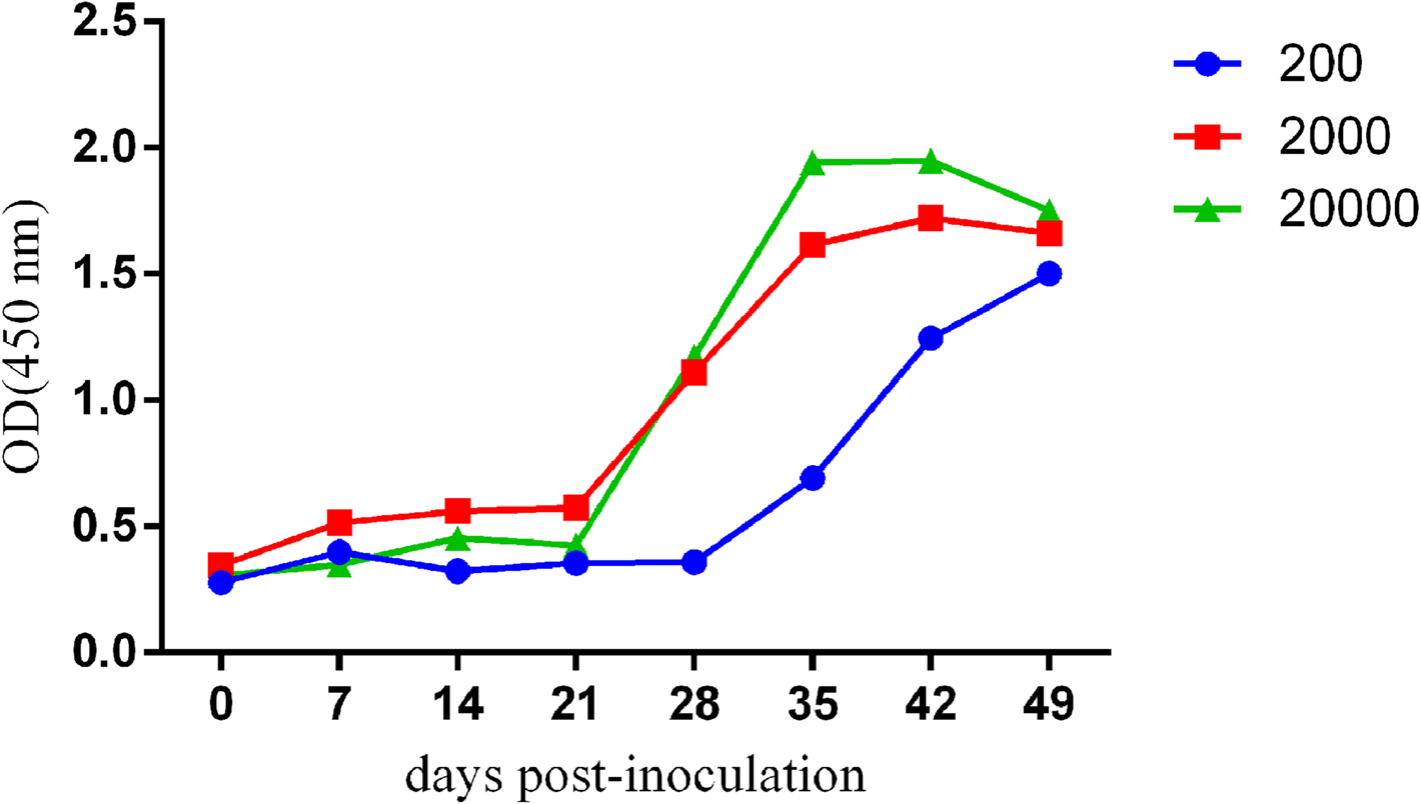
Anti-*Trichinella*-IgG kinetics in Tibetan pigs infected with different doses of Chinese Tibet isolates of *Trichinella spiralis*

The ELISA results showed that the level of antibodies corresponded with the inoculum dose before 49 dpi, and pigs that received higher larvae dose demonstrated higher antibody levels than those that received lower dose (Fig. 1). The correlation between infective dose and the antibody level at 49 dpi was described by the equation y = 0.0541ln(x) + 1.227 (R^2^ = 0.975). The level of antibodies in pigs that received 2000 and 20,000 ML increased sharply after 21 dpi, while pigs that received 200 larvae remarkably increased after 28 dpi. After 28 dpi, the level of anti-*Trichinella*-IgG in all infected pigs demonstrated a gradual increase.

### Quantification of cytokines in sera of infected pigs

**Fig. 2:**
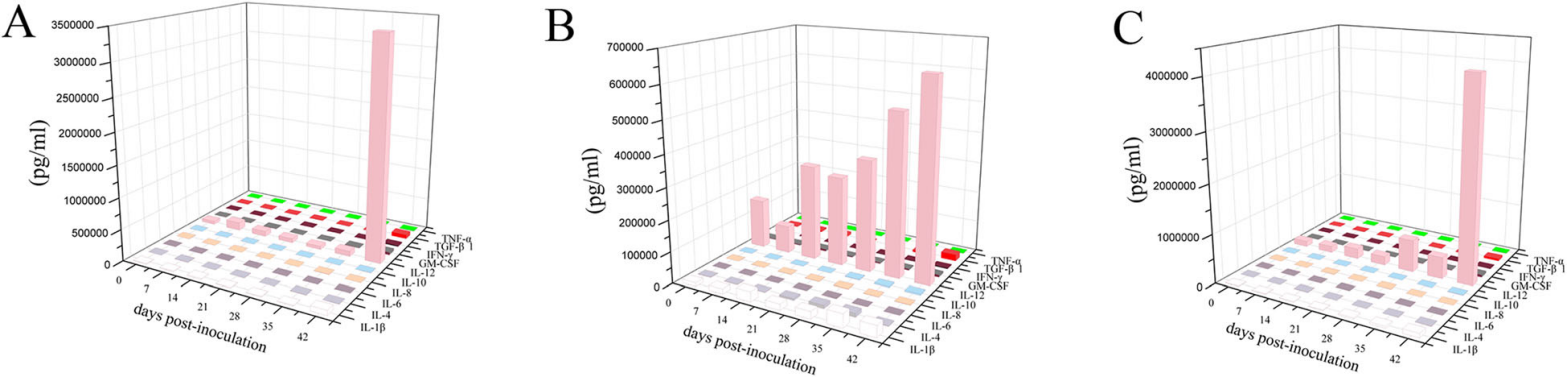
The overview of the cytokines dynamics in Tibetan pigs infected with 200 (**a**), 2,000 (**b**) and 20,000 (**c**) muscle larvae (ML) of Chinese Tibet isolate of *Trichinella spiralis*

Ten cytokines were detected by microarray in the serum samples, including four Th1 cytokines (IFN-γ, IL-1β, IL-8, IL-12), three Th2 cytokines (IL-4, IL-6, IL-10), two Th1/2 cytokines (GM-CSF, TNF-α), and one Th3 cytokine (TGF-β1). Compared with other cytokines, IL-12 demonstrated the highest concentration and peaked at 42 dpi. All cytokines showed an increasing level with days of infection, but were generally characterized by low concentrations in the early period of infection and reached a highest level at the 42 dpi (Fig. 2). Th1 cytokines and Th3 cytokines reached a higher level compared with Th2 cytokines at 42 dpi. The result indicated that the Th1 bias mixed cytokines were induced by *T. spiralis* infection in Tibetan pigs. However, the increased levels of cytokine production were irrespective of the infection dose. The levels of most cytokines in pigs inoculated with 2000 larvae were lower than those in pigs inoculated with 200 or 20,000 larvae. Meanwhile, some cytokines demonstrated higher levels in pigs receiving 200 larvae compared to those receiving 20,000 larvae.

### Seroprevalence of *Trichinella* infection in field Tibetan pigs

To estimate the prevalence of *Trichinella* in naturally infected Tibetan pigs, we collected 41 serum samples from 3 areas of Gansu province. The ELISA result showed that 18 out of the 41 samples (43.9 %) were seropositive to *Trichinella* ES antigen.

## Discussion

The first occurrence of human trichinellosis was reported in Tibet in 1964 [12, 13], and *T. spiralis* was isolated from naturally infected Tibetan pigs in Naqu Prefecture of Tibet Autonomous Region [14]. Since then, no further study has been conducted on *Trichinella* infection in Tibetan pigs. Here, we first showed that *T. spiralis* could induce high levels of specific IgG antibodies as well as Th1/Th2 mixed cytokines in Tibetan pigs. Also, the average lpg for Tibetan pigs infected with 200 ML, 2,000 ML and 20,000 ML doses of *T. spiralis* was found to be 3.5, 50.4 and 115.6, with the highest-burden lodged in the diaphragm and tongue. The average lpg for Tibetan pigs infected with 200 ML of *T. spiralis* was higher than that for Large White pigs infected with the same number of ML (0.17) [15].

Previous studies demonstrated that *T. spiralis* could elicit a predominant Th1 response at the initial intestinal phase and subsequently converts into Th2 type response [11]. The well-characterized type 2 cytokine response is beginning at the adults orchestrating niches in intestinal epithelial cells and persists during the whole chronic infection [16–18]. The Th2 immune response is characterized by the production of high levels of IL-4, IL-5, IL-9, and IL-10 [19], where IL-4 has been suggested to provide support for nematode growth through the production of IgE and tissue injury [20]. In our result, the Chinese Tibetan *T. spiralis* isolate induced an increase IL-4 production which reached its highest level at 11951.9 pg/mL after infection with 2,000 ML at 35 dpi (Fig. 2). Also, IL-6, a pleiotropic cytokine with pro- or anti-inflammatory properties [21], when deficient in mice has been found to result in worm expulsion with stronger Th2 responses, indicating that the IL-6 may promote host susceptibility against helminth infection by limiting Th2 response [22]. Macrophage, on the other hand, can be elicited to the alternative phenotype by ES products from different stages of *T. spiralis* through the inhibition of IL-6 and other proinflammatory cytokines [23]. IL-10 and TGF-β can downregulate the regional inflammation response during the intestinal and muscular stages of *Trichinella* infection [24, 25]. Meanwhile, the increased production of IL-4, IL-10 and TGF-β has been shown to modulate encephalomyelitis (EAE) [26]. In the present study, the IL-6, IL-10 and TGF-β productions varied in their levels of increase at different days post-infection, which is similar to the observation made in a study on *T. spiralis* infection in large white pigs [27]. The Th1 type cytokines were also enhanced after the infection in Tibetan pigs under the present experimental condition. The cytokine IL-12 demonstrated the highest level of increase with a concentration of about 4 × 10^6^ pg/mL. Although a mixed Th1/Th2 response can be generated from T-cell in vivo, induced by transfer of ES L1-pulsed 1DCs [28], we cannot conclude that the observed mixed Th1/Th2 response, seemingly a Th1 predominance, was induced by *T. spiralis* infection in Tibetan pigs, even though we designed our experiment to ensure accuracy and credibility by replicating the field environment of Tibetan pigs as best as possible, such that the pigs were bred under the semi-free-range system and were fed on complete formula plus Juema (*Potentilla anserina L.*). Thus, whether the marginally higher Th1 type cytokines were induced by *T. spiralis* infection or as a result of environment influence (another pathogen infection or a function of Juema) remains a subject of future investigation.

Although with limited sample size, the seroprevalence result of *Trichinella* in Tibetan pigs was 43.9 %, indicating high exposure to *Trichinella*. A similar alarming prevalence rate (33.3 %) of *Trichinella* infection in pigs was also reported in a migrant community in Delingha, Qinghai province. The pigs were raised in a free-range farming system and most of them were slaughtered domestically without inspection [9, 29]. This free-range practice where pigs feed in open fields could be responsible for the high seroprevalence of *Trichinella* infection in Tibetan pigs because they often spend long hours in the mountains during grazing, which make them suitable and potential hosts that can facilitate *Trichinella* transmission from sylvatic to domestic environments including the risk of human infection. The present study serves as a public health alert and suggests that Tibetan pigs may act as a potential risk in the spread of *Trichinella* infection, however, the prevalence of *Trichinella* infection in field Tibetan pigs warrants further investigation by pathogenic tests, such as the digestion method. Given there are no efficient special management measures for the prevention and control of *Trichinella* infection in Tibetan pigs, it is recommended that animal welfare be improved in addition to other integrated strategies to effectively control *Trichinella* infections.

## Conclusions

The present study on the immune responses of Tibetan pigs regulated by Chinese Tibet *T. spiralis* isolates demonstrated a higher level of IgG antibodies and an increased Th1/Th2 mixed cytokines. The exposure of field Tibetan pigs to *Trichinella* revealed that effective methods for the prevention and control of *Trichinella* infection in Tibetan pigs should be initiated by relevant authorities given the public health implications.

## Methods

### Animals and parasites

A total of 8 healthy female six-week-old Tibetan pigs weighing approximately 10 kg were acquired from a private source and raised *in situ* at the State Key Laboratory of Veterinary Etiological Biology, (Lanzhou, China). To confirm the absence of Infection in the Tibetan pigs, the following were investigated by ELISA, including foot and mouth disease virus (FMDV), classical swine fever virus (CSFV), porcine respiratory and reproductive disorders syndrome virus (PRRSV), porcine circovirus type 2 (PCV-2), Chlamydia, *Toxoplasma*, and some other parasite eggs by the flotation and sedimentation method. They were kept on a 12 h light/dark cycle at a temperature of 22℃, 0.1 % CO_2_ (v/v) and humidity of 60 %, with free access to food and water. Pigs were acclimated for 2 weeks (eight-week-old) before they were infected with *Trichinella*. The Chinese Tibet *T. spiralis* isolate (ISS534) was maintained in specific pathogen-free male Kunming mice in our laboratory as described previously [30]. The ML of *T. spiralis* was recovered by artificial digestion with pepsin-HCl from infected muscles at 35 dpi [31]. The correlation between lpg and the infective dose was analyzed by linear regression using the latter as a variable.

### Experimental infection and muscle larval recovery

The Tibetan pigs were averagely divided into four groups [30], including one group orally administered 0.9 % NaCl solution as negative control and other groups experimentally infected with 200, 2,000, 20,000 ML of *T. spiralis* by the same route, respectively. The lowest infection dose of *T. spiralis* infection in Tibetan pigs was determined according to a previous study [32]. Blood (5 mL) were collected from the precaval vein of the pigs and the serum were separated prior to infection and at days 7, 14, 21, 28, 35, 42 and 49 post-inoculation (dpi) by centrifugation at 2000 × g for 30 min at 4℃ and stored at -20℃ until further used. At 105 dpi, all pigs were euthanized by intravenous injection of 100 mg/kG pentobarbital sodium. After that, 20 g of muscle samples from eight organs including the diaphragm, tongue, masseter, intercostal, psoas, gluteus, foreleg and hind leg were digested artificially to evaluate the muscle larvae per gram (lpg).

### Antigen preparation and detection of total IgG

Excretory/secretory (E/S) antigen from *T. spiralis* muscle larvae was prepared as previously described [33]. The total anti-*T. spiralis* antibodies in serum were determined by ELISA using the excretory/secretory antigen. The samples included sera from days 0, 7, 14, 21, 28, 35, 42 and 49. Briefly, wells of 96-well microtiter plates were coated with 100 µL (1.5 µg/mL) of ES antigen and incubated for 2 h at 37°C. All wells were blocked with 2 % (v/v) bovine albumin in carbonate buffer solution at 4°C overnight. Each well was washed three times with PBS-Tween 0.05 % (PBS-T), followed by addition of 100 µL serum samples at a dilution of 1:100 and incubated for 1 h at 37°C. After washing three times with PBST, 100 µL of horseradish-peroxidase (HRP) conjugated goat anti-mouse IgG were added to the wells at a dilution of 1:10,000 and were incubated for 1 h at 37°C. The wash step was repeated, and then 100 µL substrate solutions were added for 10 min at 37°C. The reaction was terminated immediately by adding 50 µL stop solution. The ELISA results were read by the Model 680 Microplate reader (Bio-Rad) at 450 nm optical density (OD). The results were analyzed by the Microplate Master™ software. The correlation between the infective dose and the antibody level at 49dpi was analyzed by natural logarithm regression using the latter as a variable.

### Cytokines quantification in serum

Ten different cytokines were evaluated in the serum of 42 blood samples at days 0, 7, 14, 21, 28, 35, and 42 dpi by Quantibody Porcine Cytokine Array following the manufacturer’s instructions (Raybiotech, USA). The cytokines include four Th1 cytokines (IFN-γ, IL-1β, IL-8, IL-12), three Th2 cytokines (IL-4, IL-6, IL-10), two Th1/2 cytokines (GM-CSF, TNF-α), and one Th3 cytokine (TGF-β1). Pre-infection blood samples were used as controls.

### Seroepidemiological surveys and statistical analysis

To investigate the prevalence of *Trichinella* infection in field Tibetan pigs, a total of 41 serum samples from Tibetan pigs were examined by ELISA using ES antigen from *T. spiralis* muscle larvae. The Tibetan pigs were selected from Shuangcha township, Luqu county and Gannan Tibetan Autonomous Prefecture of Gansu province, Northwestern China. All blood samples were collected from the precaval vein of the pigs, and the sera were separated by centrifugation at 1500 × g for 20 min in the State Key Laboratory of Veterinary Etiological Biology, Lanzhou Veterinary Research Institute, Chinese Academy of Agricultural Sciences. The ELISA results were read by the Model 680 Microplate reader (Bio-Rad) using OD450 nm and analyzed by Microplate Master™ software. The results were evaluated as sample/negative control (S/N) ratio by comparing the mean OD value of the duplicate sample using Excel. An S/N ratio of ≥ 2.1 was regarded as positive.

## Data Availability

The datasets used and/or analyzed during the current study are available from the corresponding author on reasonable request.

## Abbreviations

IFN-γ: Interferon-γ
IL: Interleukin
CSF: Colony Stimulating Factor
TNF: Tumor necrosis factor
TGF: Transforming growth factor
Th1/Th2: T helper type 1/T helper type 2
ELISA: Enzyme linked immunosorbent assay
PBS: Phosphate buffer saline
